# Magnitude, Preventability, and Determinants of Adverse Drug Reactions Among Hospitalized Obstetric Patients in Ethiopia: A Prospective Observational Study

**DOI:** 10.1002/prp2.70248

**Published:** 2026-04-25

**Authors:** Getachew Yitayew Tarekegn, Endalemaw Tsegaw, Fisseha Nigussie Dagnew, Sisay Sitotaw Anberbr, Tilaye Arega Moges, Samuel Berihun Dagnew, Samuel Agegnew Wondm, Tigabu Eskeziya Zerihun, Abel Temeche Kassaw, Desalegn Addis Mussie

**Affiliations:** ^1^ Department of Pharmacy, College of Health Sciences Debre Tabor University Debre Tabor Ethiopia; ^2^ Department of Pharmacy, College of Medicine and Health Sciences University of Gondar Gondar Ethiopia; ^3^ Department of Clinical Pharmacy, Pharmacy Education and Clinical Services Directorate, College of Health Sciences Debre Tabor University Debre Tabor Ethiopia

## Abstract

Adverse drug reactions (ADRs) are an important and potentially preventable cause of morbidity among hospitalized obstetric patients, with implications for both maternal and fetal safety, yet evidence from low‐resource settings remains limited. This prospective observational study, conducted from February to May 2025 at Debre Tabor Comprehensive Specialized Hospital, Ethiopia, aimed to determine the magnitude, characteristics, preventability, and determinants of ADRs among hospitalized pregnant women and postpartum women within six weeks of delivery. ADRs were identified through daily chart review and patient interviews and confirmed using the Naranjo Adverse Drug Reaction Probability Scale, while severity and preventability were assessed using the modified Hartwig and Siegel scale and the Schumock and Thornton criteria, respectively. Multivariate logistic regression was used to identify factors independently associated with ADR occurrence. Among the 354 patients included, 52 experienced at least one ADR, yielding a prevalence of 14.7%. Most ADRs were mild to severe (88.5%) and classified as definitely preventable (82.7%). Polypharmacy (4–6 medications) was independently associated with ADR occurrence (adjusted odds ratio [AOR] = 4.02; 95% CI: 1.63–9.93). Additionally, patients with a history of adverse pregnancy outcomes (AOR = 6.28; 95% CI: 2.64–14.95) and those with underlying medical comorbidities (AOR = 2.95; 95% CI: 2.23–9.76) had significantly higher odds of experiencing ADRs. ADRs were relatively common among hospitalized obstetric patients, and the predominance of preventable events highlights the need for strengthened pharmacovigilance systems and targeted medication safety interventions to reduce preventable harm and improve maternal and fetal outcomes in resource‐limited settings.

**Abbreviations**
ADR: Adverse Drug Reaction
AOR: adjusted odds ratio
CI: confidence interval
COR: crude odds ratio
DTCSH: Debre Tabor Comprehensive Specialized Hospital
HMIS: Health Management Information System
IRB: Institutional Review Board
LMICs: Low‐ and Middle‐Income Countries
LoHS: Length of Hospital Stay
NARANJO: Naranjo Adverse Drug Reaction Probability Scale
PROM: Premature rupture of membranes
RVI: respiratory viral infection
SPSS: Statistical Package for the Social Sciences

## 1 Introduction

Adverse drug reactions (ADRs) are any unintended, harmful response to a medication administered at normal therapeutic doses for the prevention, diagnosis, or treatment of disease [1]. Globally, ADRs contribute substantially to patient morbidity and mortality, accounting for approximately 5%–10% of hospital admissions and prolonged hospital stays [2, 3]. These represent a major challenge to healthcare systems worldwide, with significant economic and clinical consequences. Despite increases, ADRs remain underreported and underrecognized, especially in vulnerable populations, such as pregnant and postpartum women.

In the field of obstetrics and gynecology, pharmacotherapy is integral to managing pregnancy‐related complications, labor, delivery, and postpartum care. However, physiologic changes during pregnancy, including altered gastrointestinal motility, plasma volume expansion, hepatic metabolism, and renal clearance, can significantly modify absorption, distribution, metabolism, and elimination, leading to altered pharmacokinetics and pharmacodynamics. These changes increase the susceptibility of pregnant women to ADRs, which may adversely affect both maternal and fetal health. Additionally, ethical constraints limit clinical trials during pregnancy, resulting in a paucity of evidence‐based guidance for medication use in this population.

Globally, the prevalence of ADRs among obstetric and gynecologic patients varies widely, with reported rates ranging from 10%–30%, influenced by variations in study design, detection methods, and healthcare [4]. Studies from high‐income countries often show lower rates due to robust pharmacovigilance systems, whereas data from low‐ and middle‐income countries (LMICs) are limited [5, 6]. In Ethiopia and similar resource‐constrained settings, the burden of ADRs may be underestimated due to weak monitoring systems, limited healthcare worker training, medication shortages, and suboptimal documentation practices [7, 8]. These challenges underscore the urgency to generate context‐specific data on ADRs in obstetrics and gynecology to inform clinical practice and health policy [9]. Furthermore, the determinants of ADRs in pregnant women are multifaceted, including predictors for adverse drug reactions (ADRs) that are crucial for interventions, but limited data in Ethiopia, especially in the northwest region, hinder effective implementation. Polypharmacy, comorbidities, and healthcare system factors. This study aims to identify ADR determinants among obstetric and gynecologic patients, providing valuable evidence for clinical decision‐making and patient safety improvement.

Guided by existing research, we evaluated the frequency and predictors of adverse drug reactions (ADRs) among hospitalized obstetric patients. We hypothesized that ADRs occur frequently due to physiological changes during pregnancy and medication exposure, with polypharmacy increasing the risk through cumulative exposure and drug interactions. Additionally, comorbidities and a history of adverse pregnancy outcomes were expected to elevate ADR risk, thereby providing a framework for our investigation into these determinants, as reflected in our multivariate regression models.

## 2 Methods

### 2.1 Study Area, Period, and Design

A prospective observational study took place from February to May 2025 at Debre Tabor Comprehensive Specialized Hospital in Ethiopia, which serves around 3.5 million people and offers extensive obstetric, gynecologic, and general medical services, including emergency obstetric care, cesarean delivery, and maternal inpatient care. This study was designed and reported following the STROBE guidelines for observational studies.

### 2.2 Populations, Inclusion, and Exclusion

The study focused on hospitalized obstetric patients, specifically pregnant women and postpartum women within six weeks of delivery at Debre Tabor Comprehensive Specialized Hospital. Inclusion criteria were women aged 18 or older hospitalized for obstetric care, who consented to participate, and had complete medical records of medications. Exclusion criteria eliminated outpatient care attendees, patients with incomplete records, and those unable or unwilling to provide consent, ensuring a homogeneous obstetric patient population.

### 2.3 Sample Size and Sampling Techniques

The sample size for adverse drug reactions (ADRs) was calculated using a single population proportion formula. Since there was no similar published study in Ethiopia and consider it 50% prevalence of ADRs. Also, considering a 0.05 margin of error (d) and 95% confidence interval, *N* = the required sample size. During the study period, the baseline counts of source populations over the previous four months were recorded at 1950, as sources from the health management information system (HMIS) and the DTCSH logbook.

$$\begin{array}{l} p=\text{the proportion of ADRs}=0.5\\ 1-p=q=0.5\\ d=\text{Expected margin of error}=0.05\\ Z\;\alpha /2=95\%\;\text{confidence interval}\;\left(\mathrm{CI}\right)=1.96,\\ N=322 \end{array}$$

*N* = initial sample size using maximum sample size calculations, finally, by adding 10% non‐response rate, the final sample is 354. A consecutive sampling technique was used to assess participants. Although the study was primarily powered for prevalence estimation, this sample size was also considered sufficient for exploratory multivariate logistic regression analysis to identify the determinants of ADR occurrence.

### 2.4 Study Variables

This study focuses on the occurrence of adverse drug reactions (ADRs) among hospitalized obstetric patients, defined by the World Health Organization as harmful responses to drugs at standard doses. ADRs included in the study were categorized as possible, probable, or definite based on the Naranjo Adverse Drug Reaction Probability Scale. Independent variables analyzed encompass sociodemographic factors (age, marital status, residence, educational level), obstetric and clinical factors (gravidity, parity, history of adverse pregnancy outcomes, comorbidities, gestational age at admission), medication‐related factors (number of medications prescribed, drug classes used, route of administration), and system‐related factors (monitoring practices and length of hospital stay).

### 2.5 Data Collection Tools and Techniques

The study collected data through a comprehensive approach, including literature review [10, 11, 12], systematic daily chart reviews, and structured interviews with patients, their families, and healthcare professionals to ensure thorough and accurate information gathering. The instrument is organized into five parts: Sociodemographic characteristics, clinical information, medication information, Naranjo Adverse Drug Reaction Probability Scale, and Schumock and Thornton criteria.

The Naranjo Adverse Drug Reaction Probability Scale is a tool used to determine the causal relationship between a clinical event and a drug using a simple questionnaire. The ADR Probability Scale consists of 10 questions that are answered as either yes, no, or “Do not know.” Different point values (−1, 0, +1, or + 2) are assigned to each answer. The actual ADR Probability Scale form and instructions on how it is completed are provided below. Total scores range from −4 to +13; the reaction is considered definite if the score is 9 or higher, probable if 5 to 8, possible if 1 to 4, and doubtful if 0 or less [13].

The Schumock and Thornton criteria were used to evaluate the preventability of alternative dispute resolution (ADR) in various studies. It has three sections: Definitely preventable, probably preventable, and non‐preventable. Section A comprises five questions, whereas Section B has four questions. All answers are categorized as “Yes” or “No.” ADRs were “definitely preventable” if the answer was “yes” to one or more questions in Section A. If the answers were all negative, we proceeded to Section B. ADRs were “probably preventable” if the answer was “yes” to one or more questions in Section B. If the answers were all negative, we proceeded to Section C. In Section C, the ADRs were non‐preventable [14].

Hartwig's Severity Assessment Scale was established to assess the severity of ADR. Mild = levels 1 and 2, moderate = levels 3 and 4, severe = 5, 6, and 7. Data were obtained from patient medical charts, patient interviews, and direct observation [15].

ADR Trigger Tool. This tool is intended to assist surveyors in identifying, (1) the extent to which facilities have identified resident‐specific risk factors for adverse drug reactions, (2) the extent to which facilities developed and implemented systems and processes to minimize risks associated with medications that are known to be high‐risk and problem‐prone, and the medication module of the Institute for Healthcare Improvement Global Trigger Tool for measuring ADEs was used to facilitate manual chart reviews and increase detection of ADEs. The use of “triggers,” or clues, to identify adverse reactions (ARs) is an effective method for measuring the overall level of harm in a health care organization. The history of medication use before hospital admission was recorded for each patient to identify prior medications taken that could contribute to adverse drug events (ADEs).

### 2.6 Outcome Measures and Validation

The primary outcome of the study was the incidence of adverse drug reactions (ADRs) in hospitalized obstetric patients, defined by the WHO as harmful responses to drugs at therapeutic doses. Only ADRs classified as possible, probable, or definite were considered. Secondary outcomes included severity assessment via the modified Hartwig and Siegel scale, categorizing ADRs as mild, moderate, or severe, and preventability was evaluated using the Schumock and Thornton criteria. Validation involved independent reviews by two pharmacists, with discrepancies resolved through consensus to enhance reliability. The use of standardized scales ensures a robust approach to assessing ADRs, minimizing bias and improving data integrity.

### 2.7 Data Quality Assurance and Missing Data Handling

To enhance the accuracy, completeness, and reliability of the collected data in the study, various measures were implemented. Data collectors, trained clinical pharmacy professionals, received instruction on study objectives, ADR definitions, assessment tools, and ethical conduct. The data collection tool underwent pretesting with a small patient population, allowing for revisions based on feedback. Daily supervision by the principal investigator ensured consistent data collection, with cross‐checking against medical records. Suspected ADRs were assessed by two trained pharmacists, resolving discrepancies through consensus with a senior clinician. Incomplete cases were excluded from the analysis, while minor missing data were sourced from multiple references to minimize loss. Documenting the proportion of missing data contributed to transparency and reduced bias, ultimately enhancing the study's validity and reliability.

### 2.8 Data Analysis and Presentation

Data were analyzed using SPSS version 27, focusing on completeness and consistency. Descriptive analysis summarized continuous variables (age, medications) with means/SDs or medians/IQRs, while categorical variables (marital status, comorbidities, and ADR occurrence) were presented as frequencies and percentages. ADR profiles were organized in tables. Bivariate logistic regression identified variables associated with ADRs, and those with *p* < 0.25 were tested in a multivariate model. Independent predictors were determined, controlling for multicollinearity, and reporting adjusted odds ratios (AORs) with 95% confidence intervals. Model validity was assessed using the Hosmer–Lemeshow test and variance inflation factors. The results, including ADR characteristics and summaries, were clearly presented in tables and figures for transparency and reproducibility in analyzing ADRs among hospitalized obstetric patients.

## 3 Definition of Terms

An allergic reaction: Occurs when triggered by allergens to which the affected individual is allergic Sensitized [16].

Adverse drug reaction: Any unexpected, unintended, undesired, or excessive response to a medicine; it is harm directly caused by the drug at normal doses, during normal use [17].

Preventable: This refers to incidents where standard professional behavior or technique was breached, necessary precautions were not taken, or the event was preventable by modifying behavior, technique, or care [18].

Non‐Preventable: ADEs occur when no obvious breach of professional behavior or technique occurred, necessary precautions were taken, and no known alteration in method or care exists to prevent the event [19].

## 4 Results

### 4.1 Demographics and Pregnancy Characteristics

Among the 354 maternal women, the mean age was 29.56 (±5.87). Most were married 328 (92.7%) and resided in urban areas 218 (61.6%). The leading occupations were farming 106 (29.9%) and governmental employment 70 (19.8% 0). Regarding education level, 99 (28%) could not read or write, whereas 82 (23.2%) had a tertiary education. In terms of parity distribution, 187 (52.8%) more than half were multiparous, followed by primiparous 101 (28.5%) and nulliparous 66 (18.6%). Most pregnancies were in the third trimester 258 (84.87%), with fewer in the second trimester 34 (11.18%) (Table 1).

**TABLE 1:** Sociodemographic, pregnancy stage, and health‐related characteristics of pregnant women at Debre Tabor Comprehensive Specialized Hospitalized from February to May 2025 (*n* = 354).

Variable	Category	Frequency (%)
Marital status	Divorced	5 (1.4)
	Married	328 (92.7)
	Single	17 (4.8)
	Widowed	4 (1.1)
Age means (SD) = 29.56 (±5.87)		
LoHS mean (SD) = 2.63 (±1.88)		
Residence	Rural	136 (38.4)
	Urban	218 (61.6)
Occupation	Daily labor	23 (6.5)
	Farmer	106 (29.9)
	Merchant	33 (9.3)
	Government employ	70 (19.8)
	Student	29 (8.2)
	Unemployed	24 (6.8)
	Wife	69 (19.5)
Educational status	Can't read and write	99 (28.0)
	Primary [1, 2, 3, 4, 5, 6, 7, 8]	80 (22.6)
	Secondary [9, 10, 11, 12]	93 (26.3)
	Tertiary (diploma and above)	82 (23.2)
Parity	Nulliparous	66 (18.6)
	Primiparous	101 (28.5)
	Multiparous	187 (52.8)
Substance used	Yes	59 (16.7)
	No	295 (83.3)
Pregnancy stage	First trimester	12 (4.0)
	Second trimester	34 (11.2)
	Third trimester	258 (84.9)

*Note:* Percentages are calculated from the total number of participants (*n* = 354). Multiparous: Women who have had at least one previous birth; primiparous: Women with no previous births. Medical comorbidities include hypertension, diabetes mellitus, anemia, and other chronic conditions.

### 4.2 Maternal Medication, Gestational Age, and Pregnancy Outcomes

This study reported that a majority of 280 (79.1%) patients had no past medication before admission, while 60 (16.9%) reported two or more medications. Gestational age was the predominant term in 178 (50.3%) term pregnancies and 164 (46.3%). Adverse pregnancy outcomes were reported in 130 (36.7%) of current pregnancies and 96 (27.1%) of previous pregnancies. The most comorbid conditions include sepsis and pneumonia (Table 2).

**TABLE 2:** Maternal medication history, gestational age, comorbid condition, and adverse pregnancy outcomes at Debre Tabor Comprehensive Specialized Hospitalized from February to May 2025 (*n* = 354).

Variable	Categories	Frequency (%)
No. of medicine taken before admission	No past medication	280 (79.1)
	Only one Past medication	14 (4.0)
	Two or more past medications	60 (16.9)
Gestational age	Post‐term pregnancy	12 (3.39)
	Preterm pregnancy	164 (46.3)
	Term pregnancy	178 (50.3)
Adverse pregnancy outcome in the current pregnancy	Yes	130 (36.7)
	No	224 (63.3)
Adverse pregnancy outcome in the previous pregnancy	Yes	96 (27.1)
	No	258 (72.9)
Co‐morbid condition	Sepsis	12 (3.4)
	Pneumonia	10 (2.8)
	UTI	6 (1.7)
	Dyspepsia	3 (0.9)
	T1 DM	3 (0.9)
	Hepatitis	2 (4.9)
	Asthma	2 (4.9)
	RVI	1 (2.4)
	Other*	2 (4.9)

*Note:* Causality was assessed using the Naranjo Adverse Drug Reaction Probability Scale. Severity was classified according to the modified Hartwig and Siegel scale: Mild, moderate, and severe. Preventability was assessed using the modified Schumock and Thornton criteria. Percentages are based on the total number of patients with ADRs (*n* = 52). Some patients experienced multiple ADRs; total ADR events = 58.

Abbreviation: ADR = Adverse Drug Reaction.

* Other comorbid conditions include infrequently reported diagnoses observed in a small number of patients (< 5%), which were grouped together due to their low individual frequencies (hepatitis, asthma, renal disease, or other documented conditions).

### 4.3 Distribution of Current Pregnancy Diagnosis

**FIGURE 1:**
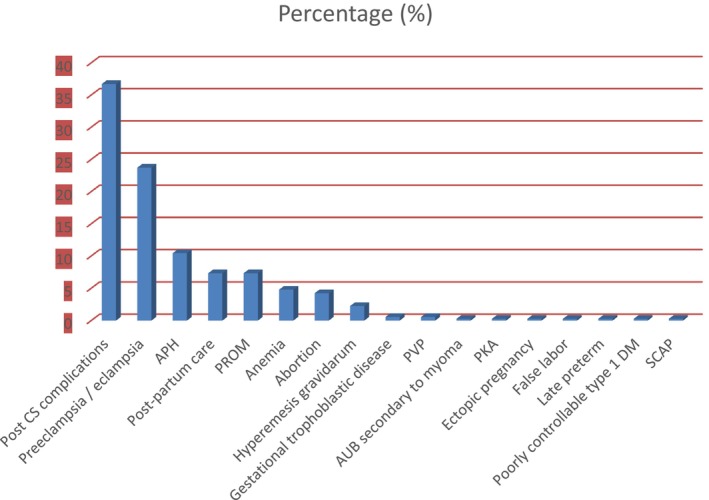
Frequency of current pregnancy‐related diagnosis among pregnant women, Debre Tabor Comprehensive Specialized Hospitalized from February to May 2025 (*n* = 354). Percentages indicate the proportion of patients within each diagnostic category. APH = Antepartum hemorrhage; AUB = abnormal uterine bleeding; DKA = diabetic ketoacidosis; PROM = Premature rupture of membranes; CS = Cesarean section; T1 DM = Type 1 diabetes mellitus; SCAP = severe community‐acquired pneumonia; JVP = Jugular venous pressure abnormality. Multiple diagnoses per patient were not considered; each patient counted once in their primary diagnosis. Categories with < 1% frequency are labeled but may appear small on the bar chart. “Pre‐eclampsia/eclampsia” includes both pre‐eclampsia and eclampsia cases. Data are derived from hospital admission records and patient clinical assessments during February–May 2025.

This study records that the most common diagnosis was post‐cesarean section complications, accounting for 120 (36.72%), followed by preeclampsia/eclampsia, 78 (23.73%). Antepartum hemorrhage was diagnosed in 34 (10.45%), while post‐partum care, premature rupture of membranes (PROM) accounted for 24 (7.34%) (Figure 1).

### 4.4 Maternal Prescribed Medications

This study reported that most of the medication history before admission showed that 280 (79.1%) had no past medication, 14 (4.0%) had one past medication, and 60 (16.90%) had two or more. During admission, the most frequently prescribed medications were diclofenac 148 (41.8%), ampicillin 114 (32.2%), ceftriaxone 112 (31.6%), and dexamethasone 97 (26.3%) (Table 3).

**TABLE 3:** Pattern of maternal medication history and prescribed treatment among maternal women at Debre Tabor Comprehensive Specialized hospital from February to May 2025 (*n* = 354).

Medication prescribed	Frequency (%)
Diclofenac	148 (41.8)
Ampicillin	114 (32.2)
Ceftriaxone	112 (31.6)
Dexamethasone	97 (26.3)
Metronidazole	55 (15.5)
Magnesium sulfate	51 (14.4)
Hydralazine	50 (14)
Tramadol	44 (12.4)
Methyl dopa	35 (9.9)
Ferrous sulfate	26 (7.3)
Amoxicillin	25 (7.1)
Erythromycin	21 (5.9)
Cimetidine	20 (5.6)
Nifedipine	16 (4.5)
Blood product	14 (4.0)
Paracetamol	14 (4.0)
Misoprostol	11 (3.1)
Metoclopramide	10 (2.8)
Oxytocin	6 (1.7)
Lasix	5 (1.4)
Other*	14 (4.0)

*Note:* Percentages are based on the total number of participants (*n* = 354); a patient may have received more than one medication, so totals may exceed 100%. “Other” includes medications prescribed in fewer than 5 patients, e.g., antiemetics, antihypertensives, or rare obstetric medications. All medications were administered according to hospital protocol and clinical indications.

* Other medications include drugs prescribed to fewer than five patients that were not listed individually, such as antiemetics, additional antihypertensives, analgesics, and other supportive obstetric medications based on clinical indication.

### 4.5 Adverse Drug Reactions by NARANJO

In this study, adverse drug reactions were observed in 52 (14.7%) of the study participants. Among the most common types of adverse drug reactions observed, 31 (59.6%) required intervention. Additionally, ADR observed 33 (63.5%) were possible, followed by Doubtful 18 (34.6%). The Naranjo questionnaire revealed a strong temporal association with adverse events, with 48 (92.3%) occurring after drug administration. However, only 22 (42.3%) patients showed improvement upon discontinuation. Challenge information was limited, and alternative causes were ruled out in 34 (65.4%). Common adverse drug reactions were side effects requiring intervention, allergic reactions, and overdose (Table 4).

**TABLE 4:** Naranjo adverse drug reaction probability and ADR types at Debre Tabor Comprehensive Specialized Hospital from February to May 2025 (*n* = 52).

Category	Frequency *n* (%)		
Scale	Yes	No	Don't know
Are there previous conclusive reports on this reaction?	42 (80.77)	10 (19.2)	
Did the adverse event appear after the suspected drug was administered?	48 (92.3)	4 (7.7)	
Did the adverse drug event improve when the drug was discontinued or a specific antagonist was administered?	22 (42.31)	2 (3.9)	28 (53.9)
Did the adverse drug event re appear when the drug was re administered?	4 (7.84)	6 (11.8)	41 (80.4)
Are there Alternative causes that could on their own have caused the reaction?	18 (34.62)	34 (65.4)	
Did the reaction reappear when a placebo was given?			52 (100)
Was the drug detected in the blood or other fluids in concentrations known to be toxic?			52 (100)
Was the reaction more severe when the dose was increased or less severe when the dose was decreased?	5 (9.6)	1 (1.9)	46 (88.5)
Did the patient have a similar reaction to the same or similar drugs in any previous exposure?	7 (13.5)	5 (9.6)	40 (76.9)
Was the adverse event confirmed by any objective evidence?	10 (19.6)	39 (76.5)	2 (3.9)
NARANJO probability score	Doubtful	18 (34.6)	
	Possible	33 (63.5)	
	Probable	1 (1.9)	
The adverse drug reactions occurred.	52 (14.7)	302 (85.3)	
The type of ADR			
Side effect requiring intervention	31 (59.6)		
The allergic reaction	20 (36.5)		
Over dose	1 (1.9)		

*Note:* Percentages for Naranjo responses and ADR type are based on ADR cases (*n* = 52); percentages for ADR occurrence are based on all study participants (*n* = 354). Naranjo probability score interpretation: Definite: ≥ 9 Probable: 5–8 Possible: 1–4 Doubtful: ≤ 0. “Don't know” indicates information not available from the chart review or patient interview. “Side effect requiring intervention” includes ADRs that require dose adjustment, therapy, or additional monitoring. Some patients experienced multiple ADRs; totals may not sum to 100%.

Abbreviation: ADR = Adverse Drug Reaction.

### 4.6 Characteristics and Severity Grades

The current study reported that most of the participants, 29 (55.8%), did not require any change in treatment. Additionally, 20 (38.5%) study participants required stopping or changing the drug but did not require any additional treatment. No ADRs that caused permanent harm or death were reported. The majority of ADRs were mild, accounting for 46 (88.7%) study participants, while a smaller proportion, 6 (11.5%), were classified as moderate in severity (Table 5).

**TABLE 5:** Characteristics and severity of adverse drug events at Debre Tabor Comprehensive Specialized Hospital from February to May 2025 (*n* = 52).

Variable	Categories	Frequency (%)
The ADR requires no change in treatment with the suspected drug.		29 (57.8)
The ADR requires the suspected drug to be withheld, discontinued, or otherwise changed. No antidote or other treatment is required. There is no increase in the length of hospital stay.		20 (38.5)
The ADR requires that the suspected drug be withheld, discontinued, or otherwise changed, and an antidote or other treatment is required. There is no increase in the length of hospital stay.		3 (5.8)
Level 4a: Any level 3 ADR that increases the length of hospital stay by at least one day.		
Any level 4 ADR that requires intensive medical care.		
ADR causes permanent harm to the patient.		
The ADR either directly or indirectly leads to the death of the patient.		
Severity grades	Mild	46 (88.7)
	Moderate	6 (11.5)

*Note:* ADR = adverse drug reaction; data are from 52 ADR cases among hospitalized obstetric patients. Clinical impact categories are based on the modified Hartwig and Siegel severity assessment scale. Level 4a–Level 7 ADRs (requiring intensive care, causing permanent harm, or death) were not observed in this study. Severity grades: Mild: No intervention or minimal intervention required. Moderate: Requires active intervention but no permanent harm. Severe: Life‐threatening or causing permanent harm (none observed). Percentages are calculated based on total ADR cases (*n* = 52).

### 4.7 Preventability and Characteristics of Adverse Drug Reactions

This study reported 52 adverse drug events (ADEs). Among these, 7 (13.5%) involved a history of allergy, 5 (9.6%) were related to inappropriate drug use, and 30 (57.7%) involved known adverse drug reactions. Notably, the majority of adverse drug reactions (43 (82.7%)) were preventable, while the remaining 9 (17.3%) were considered probably preventable according to the modified Shamrock and Thornton scales (Table 6).

**TABLE 6:** Characteristics and preventability of adverse drug events at Debre Tabor Comprehensive Specialized Hospital from February to May (*n* = 52).

Variables	Categories	Frequency (%)
Was there a history of allergy or previous reactions to the drug?		7 (13.5)
Was the drug involved inappropriate for the patient's clinical condition?		5 (9.6)
Was the dose, route, or frequency of administration inappropriate for the patient's age, weight, or disease state?		1 (1.9)
Was a toxic serum drug concentration (or laboratory monitoring test) documented?		0
Was there a known treatment for adverse drug reactions?		30 (57.7)
Was required therapeutic drug monitoring or other necessary laboratory tests not performed?		3 (5.8)
Was a drug interaction involved in the ADR?		3 (5.8)
Was poor compliance involved in the ADR?		2 (3.9)
Were preventative measures not prescribed or administered to the patient?		1 (1.9)
Preventability: Modified Shamrock and Thornton scale score	Probability preventable	9 (17.3)
	Definitely preventable	43 (82.7)

*Note:* Preventability was assessed using the modified Schumock and Thornton criteria. Percentages are based on total ADR cases (*n* = 52). “Definitely preventable” indicates that all criteria for preventability were met; “probably preventable” indicates that at least one criterion was met.

Abbreviation: ADR = Adverse Drug Reaction.

### 4.8 Factors Associated With Adverse Drug Reactions

This study identified factors associated with adverse drug events (ADEs). Patients prescribed 4–6 medications were four times more likely to experience ADEs than those prescribed 1–3 medications (AOR = 4.0; 95% CI: 1.6%–9.9; *p* = 0.003). Additionally, a previous history of adverse pregnancy outcomes increased the odds of ADEs sixfold (AOR = 6.3; 95% CI: 2.64–14.95; *p* < 0.001), and having a medical history was associated with nearly threefold higher odds of ADEs (AOR = 2.95; 95% CI: 2.23–9.76; *p* < 0.001) (Table 7).

**TABLE 7:** Bivariate and multivariate logistic regression analysis of adverse drug events at Debre Tabor. Comprehensive Specialized Hospital from February to May 2025 (*n* = 354).

Variable	Categories	ADRs Yes	ADRs No	COR (95% CI)	*P*‐value	AOR (95% CI)	*P*‐value
Gestational age	Preterm	29	135	1	—	1	—
	Term	22	156	0.7 (0.4–1.2)	0.2	0.6 (0.3–1.5)	0.3
	Post‐term	1	11	0.4 (0.1–3.4)	0.4	0.5 (0.0–7.6)	0.6
Number of drugs per patient	1–3	28	239	1	—	1	—
	4–6	22	59	3.2 (1.7–6.0)	< 0.001	4.0 (1.6–9.9)	0.003*
	≥ 7	2	4	4.3 (0.8–24.4)	0.1	3.4 (0.2–5.9)	0.4
Current adverse pregnancy outcomes	Yes	25	105	1.7 (1.0–3.1)	0.068	1.7 (0.7–4.1)	0.254
	No	27	197	1	—	1	—
Previous adverse pregnancy outcomes	Yes	30	66	4.9 (2.6–9.0)	< 0.001	6.3 (2.6–15.0)	< 0.001*
	No	22	236	1	—	1	—
Medical history	Yes	35	18	32.5 (15.3–68.8)	< 0.001	3.0 (2.2–9.8)	< 0.001*
	No	17	284	1	—	1	—
Comorbid condition	Yes	10	32	2.0 (0.9–4.4)	0.08	1.3 (0.4–3.9)	0.706
	No	42	270	1	—	1	—

*Note:* “1” indicates the reference categories for logistic regression. Multivariate logistic regression included variables with *p* < 0.25 in bivariable analysis and those clinically relevant. Collinearity was assessed (VIF < 2 for all variables), and model fit was confirmed using the Hosmer–Lemeshow goodness‐of‐fit test (*p* = 0.78). ADR prevalence is reported per patient; patients with multiple ADRs are counted once in this analysis.

Abbreviations: ADR = adverse drug reaction; COR = crude odds ratio; AOR = adjusted odds ratio; CI = confidence interval.

* Statistically significant at *p* < 0.05.

### 4.9 ADRs By Clinical Condition and Drug in Obstetric Patients

Analysis of adverse drug reactions (ADRs) indicates that among hospitalized obstetric patients, ceftriaxone and metronidazole frequently cause ADRs linked to conditions such as postoperative complications (POS CS), antepartum hemorrhage (APH), and abnormal uterine bleeding (AUB). Magnesium sulfate and oxytocin were associated with ADRs in preeclampsia and uterine atony, whereas insulin‐related ADRs were noted in diabetic ketoacidosis (DKA). Of the total ADRs observed, 18 were deemed preventable, mainly involving ceftriaxone, magnesium sulfate, and oxytocin, and most were predictable based on pharmacologic profiles or patient history. The severity of ADRs ranged from mild to moderate, with a few severe cases necessitating intensive monitoring. This analysis enhances the understanding of drug‐specific risks in obstetric care and identifies areas for targeted intervention: Table 8.

**TABLE 8:** Distribution of adverse drug reactions by drug, clinical condition, preventability, and severity among hospitalized obstetric patients (*n* = 354).

Drug/therapeutic class	Number of ADRs	Associated Condition (POS CS, APH, AUB, DKA, SCAP, etc.)	Preventability	Predictability	Severity (Mild/Moderate/Severe)
Ceftriaxone (Antibiotic)	8	POS CS, SCAP	Yes	Yes	2/5 / 1
Metronidazole (Antibiotic)	5	APH, AUB	No	Yes	3/2 / 0
Magnesium Sulfate (Anticonvulsant)	6	POS CS, Preeclampsia	Yes	Yes	1/4 / 1
Oxytocin (Uterotonic)	7	POS CS, AUB	No	Yes	2/4 / 1
Insulin (Antidiabetic)	4	DKA	Yes	Yes	1/2 / 1
Furosemide (Diuretic)	3	Heart failure	Yes	No	0/2 / 1
Hydralazine (Antihypertensive)	2	POS CS, Preeclampsia	Yes	Yes	0/1 / 1
Total/other drugs	10	Various	Mixed	Mixed	Mixed

*Note:* Preventable ADRs could have been avoided with appropriate monitoring or guideline adherence. Predictable ADRs are known from drug properties or prior patient history. Severity classification: Mild = no intervention required, moderate = requires treatment/prolongs stay, severe = life‐threatening or ICU admission. Drugs are linked to the patient's underlying condition to illustrate context‐specific risk. Data are rounded to the nearest whole numbers for clarity.

## 5 Discussion

This prospective observational study evaluated the prevalence, characteristics, and determinants of adverse drug reactions (ADRs) among obstetrics and gynecology inpatients at Debre Tabor Comprehensive Specialized Hospital (DTCSH). Approximately 15% of patients experienced ADRs, with the most classified as mild (82.69%) and deemed definitely preventable. The most common ADRs involved side effects requiring clinical intervention and allergic reactions. Key factors significantly associated with increased ADR risk included polypharmacy, a history of adverse pregnancy outcomes, and the presence of medical comorbidities. These findings underscore the critical need for targeted interventions to enhance medication safety and optimize maternal‐fetal outcomes in this patient population. This study contributes context‐specific clinical pharmacovigilance evidence from a resource‐limited setting, with implications for medication safety practice and ADR prevention in hospitalized obstetric populations.

### 5.1 Prevalence and Pattern of ADRs

The observed ADR prevalence of 14.67% in this study is consistent with prior research conducted in similar settings, such as Brazil (10.7%) and India (10.3%), and closely matches findings from a Mexican cohort reporting 12.1% [2, 20, 21, 22]. In contrast, studies from high‐income countries, e.g., a French cohort reporting only 0.3% ADRs prevalence, tend to show significantly lower rates [3]. These differences are likely due to variations in pharmacovigilance infrastructure, ADR detection methods, and reporting practices. Additionally, the higher prevalence observed here may reflect improved ADR detection techniques and differences in patients' demographics and clinical conditions. Regarding the severity, most ADRs were approximately 88% classified as mild, with a smaller proportion, nearly 12%, considered moderate. This pattern is similar to previous studies but shows a higher percentage of mild ADRs compared to some reports, such as the Brazilian study, where mild ADRs accounted for approximately 76% [2]. Differences in severity classification criteria, sample size, and healthcare facility capabilities may explain these variations.

### 5.2 Determinants of ADRs

Polypharmacy was identified as a robust predictor, with patients receiving 4–6 medications exhibiting a fourfold increased odds of experiencing an ADR. This finding is consistent with extensive literature emphasizing polypharmacy as a critical risk factor for ADRs due to increased potential drug interactions, pharmacokinetics, and pharmacodynamics complexities, and cumulative toxicities [4, 23, 24].

The presence of medical comorbidities was associated with ADR risk, with nearly a 3‐fold increase observed among affected patients. This risk underscores the vulnerability of patients with complex medical profiles, particularly in resource‐limited settings where comprehensive management of comorbid conditions is challenging [5, 25, 26]. These findings are consistent with previous studies highlighting the heightened susceptibility of multi‐morbid patients to medication‐related adverse reactions.

A history of adverse pregnancy outcomes was also significantly associated with an increased risk of ADRs (AOR = 6.28). However, frequently reported in the literature, this association is biologically plausible, as women with prior pregnancy complications may require more aggressive or complex pharmacotherapy regimens, thereby increasing their susceptibility to ADRs [10, 21, 27]. These differences may be due to study populations and methodologies and may explain the inconsistent documentation of this relationship in prior research.

### 5.3 Clinical and Public Health Implications

The higher proportion of preventable ADRs, approximately 83% identified in this study, highlights significant opportunities for interventions to improve medication safety in obstetrics and gynecology care. Strengthening clinical protocols, enhancing healthcare provider training on pharmacovigilance, and implementing robust ADR monitoring systems are critical measures to reduce the burden of ADRs. Special attention should be directed toward patients with polypharmacy, comorbidity, and adverse pregnancy outcomes histories to mitigate their elevated risk. Furthermore, the integration of systematic ADR assessment tools into routine clinical practices, alongside multidisciplinary collaboration, may enhance early identification and management of ADRs, ultimately improving maternal and fetal health outcomes in similar resource‐constrained settings.

Analysis of drug‐related adverse drug reactions (ADRs) associated with obstetric and medical conditions has revealed significant patterns. Notably, drugs such as ceftriaxone and oxytocin were linked to ADRs in patients undergoing postoperative cesarean sections and those with severe infections. In addition, magnesium sulfate and insulin were frequently associated with adverse reactions in patients with preeclampsia and diabetic ketoacidosis, respectively. These findings underscore how the risk of ADRs is influenced by the clinical context and therapeutic needs. This pattern aligns with broader pharmacovigilance studies, which indicate that most ADRs are predictable and preventable through improved prescribing practices and monitoring systems [28, 29]. Evidence from obstetric cohorts has similarly shown that magnesium sulfate and commonly used antibiotics are among the leading contributors to ADRs in high‐risk pregnancies [10, 21]. Our results emphasize the importance of context‐specific safety measures in obstetric care, suggesting that while severe ADRs are relatively rare, the predominance of mild to moderate reactions still has important implications for patient care and healthcare costs [30]. Overall, the emerging connection between ADRs and specific medical conditions calls for enhanced integration of pharmacovigilance into obstetric treatment protocols.

## 6 Strengths and Limitations of the Study

This study is among the first in Ethiopia to comprehensively investigate ADRs, particularly in hospitalized obstetrics and gynecology patients. The use of validated assessment tools, including the Naranjo probability scale, the Naranjo ADR Probability Scale, and the Modified Hartwig and Siegel Severity Assessment Scale, strengthened the reliability of ADR identification and severity classification. Additionally, the prospective design and systematic data collection through daily chart reviews and structured interviews enhanced data accuracy and completeness.

However, the study has some limitations. The relatively small sample size and short duration of the study may limit the generalizability of the findings to other healthcare settings or broader populations. The lack of advanced laboratory diagnostics constrained the ability to confirm certain ADRs objectively. Furthermore, potential underreporting or missed ADRs could have occurred despite rigorous monitoring, given resource constraints typical of the study setting. Future studies with larger cohorts, longer follow‐up, and incorporation of advanced diagnostic tools are warranted to validate and expand upon these findings.

## 7 Conclusion

This study demonstrated a substantial prevalence of adverse drug reactions (ADRs) among obstetrics and gynecology inpatients at DTCSH, with the majority of ADRs being mild yet largely preventable. Polypharmacy, prior adverse pregnancy outcomes, and existing medical comorbidities were identified as significant independent predictors of ADR occurrences. These findings highlight the urgent need to strengthen clinical protocols, enhance healthcare provider training, and implement robust pharmacovigilance systems to reduce preventable ADRs. Improving medication safety measures in obstetric and gynecologic care is essential to safeguard maternal and fetal health and optimize overall patient outcomes in resource‐limited settings.

## Author Contributions

**Getachew Yitayew Tarekegn:** conceptualization, methodology, investigation, data curation, formal analysis, software, visualization, writing – original draft, writing – review and editing. **Endalemaw Tsegaw:** supervision, project administration, validation, writing – review and editing. **Fisseha Nigussie Dagnew:** formal analysis, software, writing – original draft. **Sisay Sitotaw Anberbr:** investigation, project administration, writing – review and editing. **Tilaye Arega Moges:** resources, data curation, visualization, writing – review and editing. **Samuel Berihun Dagnew:** supervision, writing – review and editing. **Samuel Agegnew Wondm:** supervision, formal analysis, writing – review and editing. **Tigabu Eskeziya Zerihun:** conceptualization, supervision, writing – review and editing. **Abel Temeche Kassaw:** formal analysis, software, writing – review and editing. **Desalegn Addis Mussie:** data curation, investigation, writing – review and editing.

## Funding

The authors have nothing to report.

## Ethics Statement

Ethical clearance and approval were obtained from the institutional review board of Debre Tabor University (Ref No: 654/2024). Oral informed consent was obtained from the participants after explaining the study objective. Each participant was made aware that engagement was optional and that they could terminate the study at any time if they felt uncomfortable with the questionnaires. Omitting patient identification and code numbers maintained patient confidentiality. All rules and regulations were followed according to the Helsinki Declaration.

## Consent

All authors have read and approved the final version of the manuscript and consented to its publication. Where applicable, informed consent was obtained from all individual participants included in the study for their data and any accompanying images to be published.

## Conflicts of Interest

The authors declare no conflicts of interest.

## Data Availability

The data that support the findings of this study are available on request from the corresponding author. The data are not publicly available due to privacy or ethical restrictions.
